# Decreased absolute number of peripheral regulatory T cells in patients with idiopathic retroperitoneal fibrosis

**DOI:** 10.3389/fimmu.2022.1012513

**Published:** 2022-11-29

**Authors:** Lu Liu, Huanhuan Yan, Yanyan Wang, Yuhuan Xie, Lei Jiang, Jinfang Zhao, Chong Gao, Xiaofeng Li, Caihong Wang

**Affiliations:** ^1^ Department of Rheumatology, The Second Hospital of Shanxi Medical University, Taiyuan, China; ^2^ Shanxi Key Laboratory of Immunomicroecology, Taiyuan, Shanxi, China; ^3^ Department of Rheumatology, Third Hospital of Shanxi Medical University, Shanxi Bethune Hospital, Shanxi Academy of Medical Sciences, Tongji Shanxi Hospital, Taiyuan, Shanxi, China; ^4^ Department of Medical Statistics, Shanxi Medical University, Taiyuan, Shanxi, China; ^5^ Pathology, Joint Program in Transfusion Medicine, Brigham and Women’s Hospital/Children’s Hospital, Harvard Medical School, Boston, MA, United States

**Keywords:** idiopathic retroperitoneal fibrosis (IRPF), T helper 17 (Th17) cells, regulatory T (Treg) cells, IL-6, IL-10

## Abstract

**Objective:**

In order to determine whether the immune balance of T helper 17(Th17)/regulatory T(Treg) is related to the pathogenesis of idiopathic retroperitoneal fibrosis (IRPF), we analyzed the differences in peripheral blood lymphocytes, CD4+T cell subsets and cytokines between patients with IRPF and healthy people to clarify the CD4+T cell subsets, especially Treg cell subsets, and the role of cytokines in the pathogenesis of IRPF.

**Methods:**

This study included 22 patients with IRPF, 36 patients with IgG4-related diseases (IgG4-RD) without retroperitoneal fibrosis (RPF), and 28 healthy controls. The absolute numbers and percentage of peripheral blood lymphocyte subsets and CD4+T cell subsets in each group were detected by flow cytometry, and the serum cytokine level was detected by flow cytometric bead array (CBA).

**Results:**

Compared with the healthy group, the absolute value of B cells in peripheral blood of IRPF patients was significantly decreased, and T, natural killer (NK), CD4+ and CD8+ were not significantly abnormal. The absolute numbers of Th2 cells were lower than healthy group(p=0.043). In particular, the absolute numbers of Treg cells were significantly lower than healthy group(p<0.001), while the absolute numbers of Th17 cells increased(p=0.682). Th17/Treg was significantly higher than healthy group (p< 0.001). Cytokine analysis showed that the level of interleukin (IL)-4 in IRPF patients was higher than healthy group(p=0.011), IL-6, IL-10, IL-17, TNF-α and IFN-γ were significantly higher than healthy group (all p<0.001). Receiver operating characteristic (ROC) curves showed that IL-10 and TNF-α could distinguish bilateral ureteral dilatation in IRPF patients, with areas under the ROC curve (AUCs) of 0.813 (95% CI:0.607-1.000, p=0.026) and 0.950 (95% CI:0.856-1.000, p=0.001), respectively. IL-6 could distinguish bilateral ureteral obstruction, with an AUC of 0.861 (95% CI: 0.682-1.000, p=0.015).

**Conclusions:**

Our study showed that IRPF patients had reduced Treg cells and indeed had Th17/Treg imbalance, which may be related to the pathogenesis of the disease. The levels of IL-6, IL-10 and TNF-α appear to be associated with the progression of IRPF.

## 1 Introduction

Retroperitoneal fibrosis (RPF) is a rare fibrous inflammatory disease, which exists in the retroperitoneal tissue. It is characterized by inflammation and fibrosis in the soft tissues of the retroperitoneal and other abdominal organs. It usually involves the abdominal aorta, the outer membrane of the iliac artery and the surrounding retroperitoneum, which can cause ureteral obstruction and renal failure (1–3). The disease may be immune-mediated, such as inflammatory cell infiltration accompanied by a fibrotic response, and is associated with autoimmune diseases (4) and HLA-DRB1*03 (5).

Idiopathic retroperitoneal fibrosis (IRPF) accounts for about 70-80% of all RPF. It is a disease of unknown etiology with environmental and genetic susceptibility, while secondary diseases are usually related to malignant tumors, infections, drugs, radiotherapy or other diseases (1–3, 6). IRPF estimated that the incidence was 0.1-1.3 cases per 100,000 per year, and the prevalence was 1.4 cases per 100,000. The ratio of male to female was 2:1-3:1, and the average age of onset was between 50 and 70 years old (2, 7). IRPF has a hidden insipid, with abdominal, back or waist pain and systemic symptoms as the main symptoms, as well as edema of both lower limbs, oliguria or anuria, and testicular pain with or without scrotal swelling in men (8). In recent years, with the proposal of the concept of IgG4-related diseases (IgG4-RD), researchers have found that based on histological results, a portion of IRPF can be classified as IgG4-RD, thus IRPF can be divided into IgG4 and non-IgG4-related. At present, studies have found that the pathogenesis of IgG4-RD-related RPF and IRPF may be different (9–11). This paper mainly studies the pathogenesis of IRPF.

It is generally believed that the pathogenesis of IRPF is related to different types of immune cells. The histological feature of IRPF is a mixture of fibrous tissue and chronic inflammation. The fibrous tissue is mainly myofibroblasts in type I collagen matrix. Inflammatory infiltration is composed of a large number of lymphocytes, plasma cells and macrophages. Nodular aggregates with B cell cores are surrounded by T cells (mainly CD4+T cells), which reproduce the structure of germinal centers and reveal the formation of ectopic lymphocytes, which is a typical discovery. CD4+T cells are abundant in biopsy tissues (2, 12). CD4+T cells are divided into unique subpopulations according to the cytokines they secrete and their distinct functions: helper T cells 1 (Th1), Th2, and Th17 have pro-inflammatory effects, causing autoimmune and inflammatory reactions, while regulatory T (Treg) cells have anti-inflammatory effects, regulating immune balance, and maintaining immune tolerance (13). Although extensive and profound studies have been conducted on Th1 cells and Th2 cells before, more and more studies have found that the immune imbalance of Th17/Treg is involved in the pathogenesis of a variety of autoimmune diseases. For IRPF, there are currently no studies on the immune balance of Th17/Treg. Therefore, the study of peripheral CD4+T cells in patients with IRPF is helpful to provide a novel therapeutic strategy.

In this study, in order to determine whether the immune balance of Th17/Treg is related to the pathogenesis of IRPF, we analyzed the differences in peripheral blood lymphocytes, CD4+T cell subsets and cytokines between patients with IRPF and healthy people to clarify the CD4+T cell subsets, especially Th17 and Treg cell subsets, and the role of cytokines in the pathogenesis of IRPF.

## 2 Materials and methods

### 2.1 The patient

A total of 22 IRFP patients (patients with a definite diagnosis of IgG4-RD and elevated serum IgG4 levels were excluded) admitted to the Department of Rheumatology, the Second Hospital of Shanxi Medical University from December 2015 to October 2021 were enrolled, including 14 males and 8 females, with an average age of 59.91 ± 2.16 years. The control group included 28 gender-matched and age-matched healthy people and 36 patients with IgG4-RD without RPF(IgG4-RD-non-RPF). This study was approved by the Ethics Committee of the Second Hospital of Shanxi Medical University.

IRPF usually has no specific laboratory indicators, and is mainly diagnosed by imaging such as computed tomography (CT) or magnetic resonance imaging (MRI). The discovery of soft tissue masses surrounding the abdominal aorta and iliac artery and possibly surrounding adjacent structures such as the ureter and inferior vena cava usually suggests the diagnosis of retroperitoneal fibrosis. Histological examination of retroperitoneal tissue is usually required when a mass shows atypical location (e.g., pelvic, peripancreatic), or when other clinical or laboratory findings indicate the presence of underlying malignant disease or infection.

### 2.2 Clinical data

The medical history, laboratory data and imaging data of all patients were collected. The medical history includes age, sex, age of onset, and clinical presentation. Laboratory data included white blood cell (WBC) count, hemoglobin (HB) level, lymphocyte (LY) count, platelet (PLT) count, eosinophil (EOS) count, neutrophil (N) count, erythrocyte sedimentation rate (ESR), C-reactive protein (CRP), and renal function, complement C3 and C4,immunoglobulin (Ig)G, M, A, and autoantibodies [including rheumatoid factor antigen (RF), antinuclear antibody (ANA), and antineutrophil cytoplasmic antibody (ANCA)], thyroglobulin antibody (Tg-Ab), absolute numbers and percentage of peripheral lymphocytes, CD4+T cell subsets, and cytokine level. All blood samples required for clinical indicators were collected after fasting in the morning of the second day after admission. Imaging examinations included ultrasound, CT, enhanced CT, MRI, 18F-fluorodeoxyglucose positron emission tomography/computed tomography (PET/CT), intravenous pyelography and pathologically examined.

### 2.3 Materials and method

#### 2.3.1 Absolute numbers of peripheral blood lymphocytes

Two BD Trucount tubes (A and B) containing a known number of fluorescent beads were consecutively numbered and 50μL of fully mixed anticoagulant whole blood was added. Added 20μL anti-CD3-FITC/CD8PE/CD45PercP/CD4APC antibodies into tube A, 20μL anti-CD3-FITC/CD16+56-PE/CD45-PercP/CD19-APC antibodies were added to B tube (all from BD Biosciences). Mixed the tube contents thoroughly and incubated at 25°C, without light for 15-20 minutes. Then, 450μL 1X FACS hemolysin was added and fully mixed, and incubated at 25°C, dark for 15 minutes. Washed with phosphate buffered saline (PBS) and tested on the machine within 24 hours. For each sample, 15000 cells were obtained for analysis using BD MultiSET software (BD Biosciences).

#### 2.3.2 CD4+ T lymphocyte subset detection

**Figure 1:**
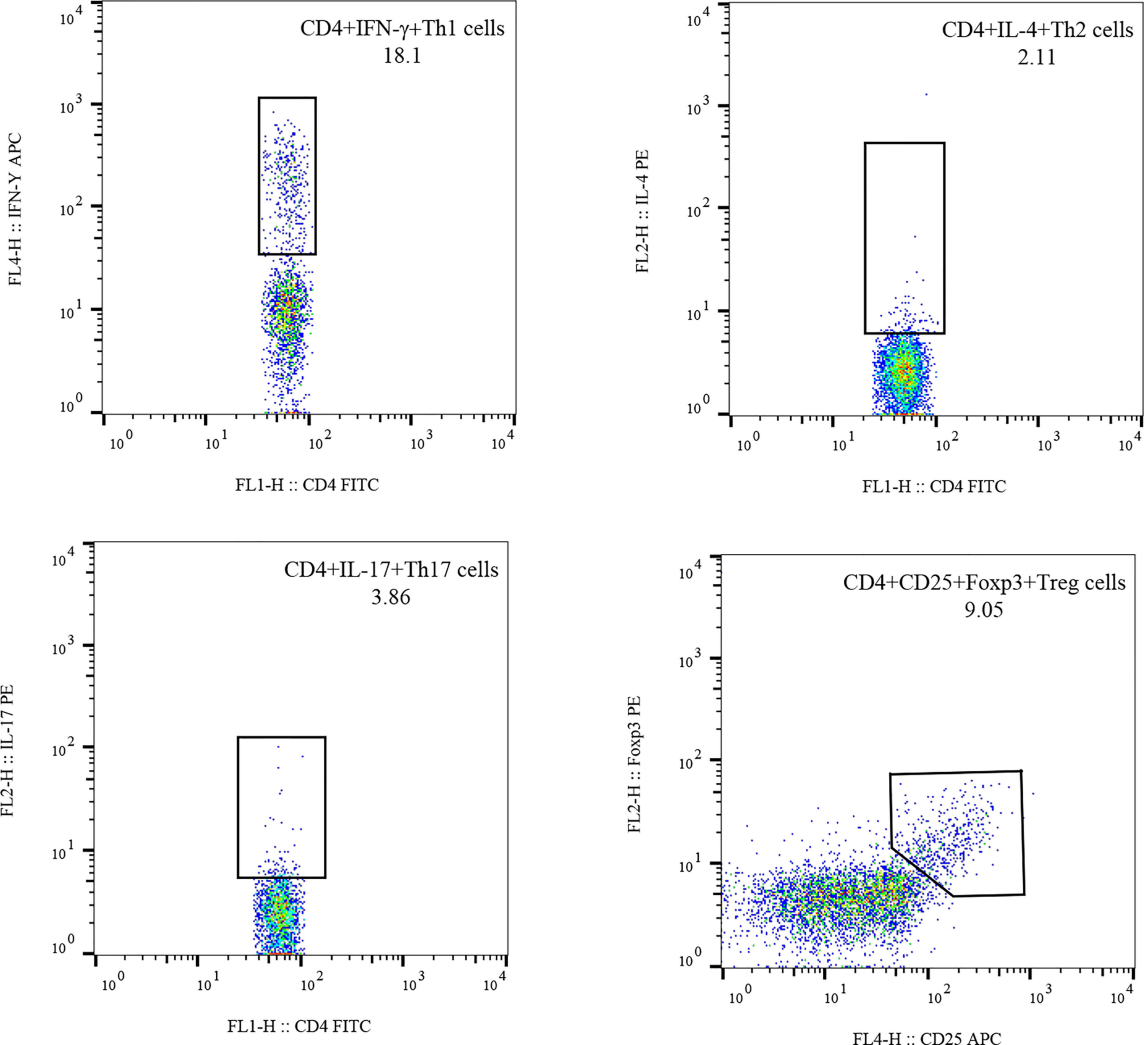
Gating for Th1,Th2,Th17 and Treg cells.

(1) Culture and labeling of Th1, Th2 and Th17 cells: 80μL anticoagulant blood was taken and added with 10μL phorbol myristate acetate working solution (final concentration 30ng/mL), 10μL ionmycin working solution (final concentration 750ng/mL) and 1μL GolgiStop at 37°C and 5% CO2 for 5h. Then, the samples were divided equally into tubes A and B, and the anti-CD4-FITC antibodies were added, and the samples were dyed at room temperature for 30 minutes without light. Then, 1ml freshly prepared fixation/permeabilization solution was added into the two test tubes, respectively, and mixed well, and incubated at 4°C under dark conditions for 30 minutes. Anti-interferon (IFN)-γ-APC and anti-IL-4-PE were added into A tube to label Th1 cells and Th2 cells, respectively. Anti-human IL-17-PE was added into B tube to label Th17 cells. The cells were stored at room temperature, away from light for 30 min, and rinsed with PBS. The absolute numbers of CD4+T lymphocyte subsets were automatically detected using BD Multitest software (BD Biosciences). All immunofluorescent antibodies were purchased from BD Biosciences.

(2) Treg cells culture and labeling: First, 80μL anticoagulant blood was added with anti-CD4-FITC and anti-CD25-APC, and incubated at room temperature for 30 minutes under dark conditions. Then, 1ml freshly prepared fixation/permeabilization solution was added into the test tube, and mixed well, and incubated at 4°C under dark conditions for 30 minutes. The anti-Foxp3 antibodies were added and incubated at room temperature in dark for 30 minutes, then washed with PBS, and detection of Treg cells using flow cytometry. All immunofluorescent antibodies were purchased from BD Biosciences.

(3) Flow cytometry: The stained cells were measured using flow cytometry (FACSCanto II; BD Biosciences) within 24 h. Based on the scatter plot of the forward angular scattered light relative to the lateral angular dispersive light (side scatter (SSC)), the lymphocytes were gated to distinguish them. CD4 was used to distinguish CD4+ T cells from the SSC gate; 10,000 cells from the gate were taken. The relative percentages were obtained and analyzed using CellQuest software. The absolute number of cells in each subgroup was calculated using the following equation: absolute cell number = percentage of positive cells in each subset × absolute number of CD4+ T cells (cells/ul) cells/ul whole blood (**Figure 1**).

#### 2.3.3 Detection of cytokine levels by cytometric bead array

Serum was separated from 4ml venous blood and stored at -20°C. Interleukin (IL)-2, IL-4, IL-6, IL-10, IL-17, IFN-γ and tumor necrosis factor (TNF)-α were detected by flow cytometry. A cytometric bead array (CBA) kit was purchased from Jiangsu Sage Biotechnology Co. Ltd. (Jiangsu, China) and used according to manufacturer’s instructions; The results were expressed as pg/ml.

### 2.4 Statistical analysis

Chi-square goodness of fit test was used for counting data. Measurement data were tested by Shapiro-Wilk test and Levene’s -T test. Normal distribution and homogeneity of variance were expressed by mean ± standard deviation and independent sample T test. Non-normally distributed data are represented as median (interquartile range). Mann-whitney U test was used for data between two groups, and Kruskal-Wallis H test was used for data between multiple groups. Spearman correlation was used for correlation analysis. The receiver operating characteristic (ROC) curve was used to detect the optimal cutoff value and validity of a variable, and the prediction effect was evaluated according to the area under the curve (AUCs). A P value (two-tailed) was considered to be less than or equal to 0.05, which was statistically significant. Statistical calculations were performed using SPSS 26.0 and GraphPad Prism 8.0 software.

## 3 Results

### 3.1 Clinical and demographic features

There were 22 patients (including 14 males and 8 females) with IRPF who met the diagnostic criteria were enrolled in this study. The demographic data and clinical characteristics were presented in **Table 1**. The mean age of IRPF patients was 59.91 ± 10.13 years, and the median time from onset to diagnosis was 2.13(1.00-15.88) months. The healthy control group included 17 males and 7 females, with an average age of 55.67 ± 12.45 years. The IgG4-RD-non-RPF group included 24 males and 12 females, with an average age of 54.92 ± 10.76 years. The main clinical characteristics of IRPF patients were low back pain (16,72.7%) and abdominal pain (9,40.9%). Other characteristics included bilateral lower limb edema (4,18.2%), oliguria or anuria (2,9.1%) and systemic symptoms including fatigue (6,27.3%), loss of appetite (5,22.7%), nausea (5,22.7%), fever (3,13.6%), and loss of weight (5,22.7%). Seven patients underwent ureterolysis, two underwent nephrostomy, and two received oral nonsteroidal drugs. Retroperitoneal histopathology was performed in 2 patients. Laboratory characteristics were shown in **Table 2**.

**Table 1:** Demographic and clinical characteristics of 22 patients with IRPF.

Items	IRPF group (n=22)
Gender (male/female)	14/8
Age (years),mean ± SD	59.91 ± 10.13
Disease duration (month), median (IQR)	2.13 (1.00-15.88)
ANA-positive	7
ANCA-positive	3
RF-positive	4
Tg-Ab-positive	7
Smoking	10
Clinical characteristics	
Low back pain (%)	16 (72.7%)
Abdominal pain (%)	9 (40.9%)
Bilateral lower limb edema (%)	4 (18.2%)
Systemic symptom	
Fatigue (%)	6 (27.3%)
Loss of appetite (%)	5 (22.7%)
Nausea (%)	5 (22.7%)
Fever (%)	3 (13.6%)
Loss of weight (%)	5 (22.7%)
Oliguria or anuria (%)	2 (9.1%)
Bilateral ureters are involved (%)	8 (36.4%)
Unilateral ureter is involved (%)	10 (45.5%)
Renal insufficiency (%)	3 (13.6%)
Kidney failure (%)	1 (4.5%)
Biopsy	2
Treatment	
Untreated	12
Nonsteroidal drugs	2
Renal colostomy	2
Ureterolysis	7

IRPF, Idiopathic retroperitoneal fibrosis; SD, standard deviation; IQR; Inter quartile range; ANA, anti-nuclear antibodies; ANCA, antineutrophil cytoplasmic antibody; RF, rheumatoid factor; Tg-Ab, thyroglobulin antibody.

**Table 2:** Characteristics and laboratory data of IRPF group and IgG4-RD-non-RPF group.

	IRPF (n=22)	IgG4-RD-non-RPF (n=36)	*P*-value
Demographics			
Age (years)^a^	59.91 ± 10.13	54.92 ± 10.76	0.085
Male,n (%)	14 (63.64%)	24 (66.67%)	0.814
Female,n (%)	8 (36.36%)	12 (33.33%)	–
Laboratory characteristics			
ESR, (mm/h)^b^	68.00 (24.75-120.00)	27.00 (15.75-51.25)	0.021*
CRP (mg/ml)^b^	14.05 (4.39-45.15)	3.14 (2.86-6.83)	0.005*
WBC (*10^9^/L)^b^	6.81 (5.79-8.72)	6.36 (5.16-7.41)	0.309
Hb (g/L)^a^	120.28 ± 20.24	129.43 ± 20.63	0.104
PLT (*10^9^/L)^a^	251.50 ± 78.67	227.45 ± 97.85	0.334
LY (*10^9^/L)^a^	1.48 ± 0.55	1.76 ± 0.62	0.082
N (*10^9^/L)^b^	4.79 (3.43-5.93)	3.48 (2.57-4.47)	0.007**
EOS (*10^9^/L)^b^	0.07 (0.03-0.16)	0.27 (0.11-0.47)	<0.001***
BUN (nmol/L)^b^	6.31 (4.58-9.97)	5.25 (3.90-6.25)	0.053
Cr (μmol/L)^b^	88.50 (69.50-124.75)	64.00 (52.25-74.75)	<0.001***
IgG (mg/dl)^b^	15.90 (10.70-18.90)	18.05 (14.15-23.65)	0.036*
IgA (mg/dl)^b^	2.81 (1.53-3.42)	1.89 (1.28-2.76)	0.059
IgM (mg/dl)^a^	0.95 ± 0.30	1.03 ± 0.53	0.472
IgG4 (mg/dl)^b^	672.50 (367.00-1042.50)	11100.00 (3612.50-18575.00)	<0.001***
IgG4/IgG^b^	43.23 (17.23-75.53)	574.66 (291.35-879.25)	<0.001***
C3 (g/L)^a^	0.90 ± 0.29	0.69 ± 0.25	0.023*
C4 (g/L)^a^	0.27 ± 0.09	0.14 ± 0.06	<0.001***

a Results are expressed as the mean ± standard deviation.

b Results are expressed as the median and 25th and 75th percentiles.

The independent-samples t test was used for analysis of quantitative variables with normal distributions. Mann-Whitney U test was used for analysis of quantitative variables with a non-normal distribution. Chi-square test was used for categorical variables.

IRPF, Idiopathic retroperitoneal fibrosis; IgG4-RD-non-RPF, IgG4-related diseases without RPF; ESR, erythrocyte sedimentation rate; CRP, C-reactive protein; WBC, white blood cell; Hb, hemoglobin; PLT, platelet; LY, lymphocyte; N, neutrophil; EOS, eosinophil; BUN, blood urea nitrogen; Cr, creatinine; Ig, Immunoglobulin; C3, Complement 3; C4, Complement 4. *P < 0.05, **P < 0.01, ***P < 0.001.

### 3.2 Decreased peripheral Treg cells and an increased Th17/Treg ratio in IRPF patients

**Figure 2:**
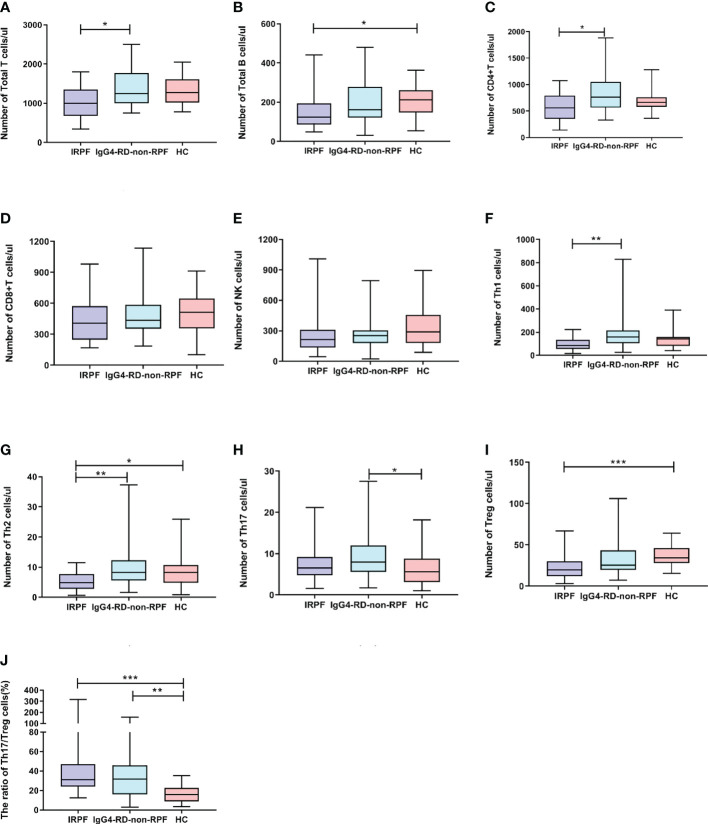
Comparison of lymphocyte absolute values and CD4+ T cell subsets IRPF group (n = 22), and IgG4-RD-non-RPF (n = 36) and healthy control group(n=28). Data were presented as mean ± SD. Shown significant differences are assessed by the Kruskal-Wallis H test. P < 0.05 was considered statistically significant. (*p < 0.05, **p < 0.01, and ***p < 0.001). **(A–E)** represent the differences in the absolute value of lymphocyte in three groups (corresponding to Total T, Total B, NK, CD4+T, CD8+T,respectively). **(F–J)** means the differences in absolute value of Th subsets of three groups (corresponding to Th1, Th2,Th17, Treg, Th17/Treg,respectively).

Peripheral blood lymphocytes and CD4+T lymphocyte subsets were detected in all 22 patients. Compared with HC, the absolute value of B cells in IRPF patients was significantly increased [124.08(86.59-193.76) vs 212.50 (148.42-262.27), p=0.018], and there was no significant difference in the absolute value of T, NK, CD4+T and CD8+T cells(**Supplementary Table 1**; **Figures 2A–E**). For CD4+T cell subsets, the absolute values of Th1 cells and Th2 cells [85.78(55.58-135.96) vs 141.75(82.73-159.18), p=0.118, and 4.88(2.87-7.68) vs 8.27(4.93-10.66),p=0.043] in IRPF patients were lower than those in HC. In particular, the absolute values of Treg cells [19.55(12.18-30.16) vs 34.55(28.20-46.38),p<0.001] in IRPF patients were significantly lower than those in HC, while the absolute values of Th17 cells were increased [6.52(4.79-9.18) vs 5.60(3.16-8.78),p=0. 0.682], so Th17/Treg(%) was significantly higher than that in healthy group, and the difference was significant [31.21(24.00-46.82) vs 15.99(8.93-22.56), p<0.001] (**Supplementary Table 2**; **Figures 2F–J**).

Compared with IgG4-RD-non-RPF, the absolute values of T cells [999.76(678.68-1352.28) vs 1251.92(996.62-1775.63), p=0.036], CD4+ cells [562.77(352.65-789.39) vs 765.12(564.34-1051.14), p=0.011], Th1 cells [85.78(55.58-135.96) vs 158.04(107.16-216.11), p=0.001] and Th2 cells [4.88(2.95-7.11) vs 8.33(2.87-7.68), p=0.004] in IRPF patients were significantly decreased. There were no significant differences in the absolute values of Th17 cells, Treg cells and Th17/Treg between IRPF patients and IgG4-RD-non-RPF patients (**Supplementary Tables 1, 2**).

### 3.3 Cytokine increased in IRPF patients

Cytokine levels were measured in 18 of 22 IRPF patients (**Table 3**). Compared with HC, the levels of IL-4 [2.89(1.42-4.36) vs 1.34(1.22-1.49), p=0.011], IL-6 [10.97(4.80-22.82) vs 2.46(2.03-3.56), p<0.001], IL-10 [5.79(4.27-8.17) vs 1.78(1.56-2.00), p<0.001], IL-17 [11.85(5.52-19.38) vs 0.73(0.27-1.33), p=0.001], IFN-γ [4.35(3.05-6.42) vs 1.39(1.20-1.68), p<0.001] and TNF-α [3.38(1.63-4.87) vs 1.16(1.06-1.41), p<0.001] in IRPF patients were significantly increased, but there was no significant difference in IL-2.

**Table 3:** Serum cytokine levels in IRPF group(A), and IgG4-RD-non-RPF(B) and Healthy control group(C).

Cytokine (pg/ml)	IRPF(A)(n=18)	IgG4-RD-non-RPF(B) (n=22)	HC(C) (n=28)	*P-*Value	*A* vs. *B*	*A* vs. *C*	*B* vs. *C*
Male,n (%)	12(66.7%)	15(68.2%)	9(32.1%)	–	–	–	–
Female,n (%)	6(33.3%)	7(31.8%)	19(69.7%)	–	–	–	–
IL-2	2.38 (1.55-3.34)	2.37 (1.18-4.85)	1.71 (1.58-1.86)	0.114	–	–	–
IL-4	2.89 (1.42-4.36)	1.88 (1.26-4.43)	1.34 (1.22-1.49)	0.006**	1.000	0.011*	0.046*
IL-6	10.97 (4.80-22.82)	11.99 (5.34-24.27)	2.46 (2.03-3.56)	<0.001***	1.000	<0.001***	<0.001***
IL-10	5.79 (4.27-8.17)	5.34 (4.03-14.50)	1.78 (1.56-2.00)	<0.001***	1.000	<0.001***	<0.001***
IL-17	11.85 (5.52-19.38)	5.56 (0.07-14.70)	0.73 (0.27-1.33)	0.001**	0.171	0.001**	0.140
IFN-γ	4.35 (3.05-6.42)	3.40 (1.92-6.38)	1.39 (1.20-1.68)	<0.001***	1.000	<0.001***	<0.001***
TNF-α	3.38 (1.63-4.87)	3.37 (1.61-7.08)	1.16 (1.06-1.41)	<0.001***	1.000	<0.001***	<0.001***

Results are expressed as the median and 25th and 75th percentiles.

Statistics: Kruskal-Wallis H test.

IRPF, Idiopathic retroperitoneal fibrosis; IgG4-RD-non-RPF, IgG4-related diseases without RPF; HC, Healthy control; IL-2, interleukin-2; IL-4, interleukin-4; IL-6, interleukin-6; IL-10, interleukin-10; IL-17, interleukin-17; INF-γ, interferon-γ; TNF-α, tumor necrosis factor-α. *P < 0.05, **P < 0.01, ***P < 0.001.

**Figure 3:**
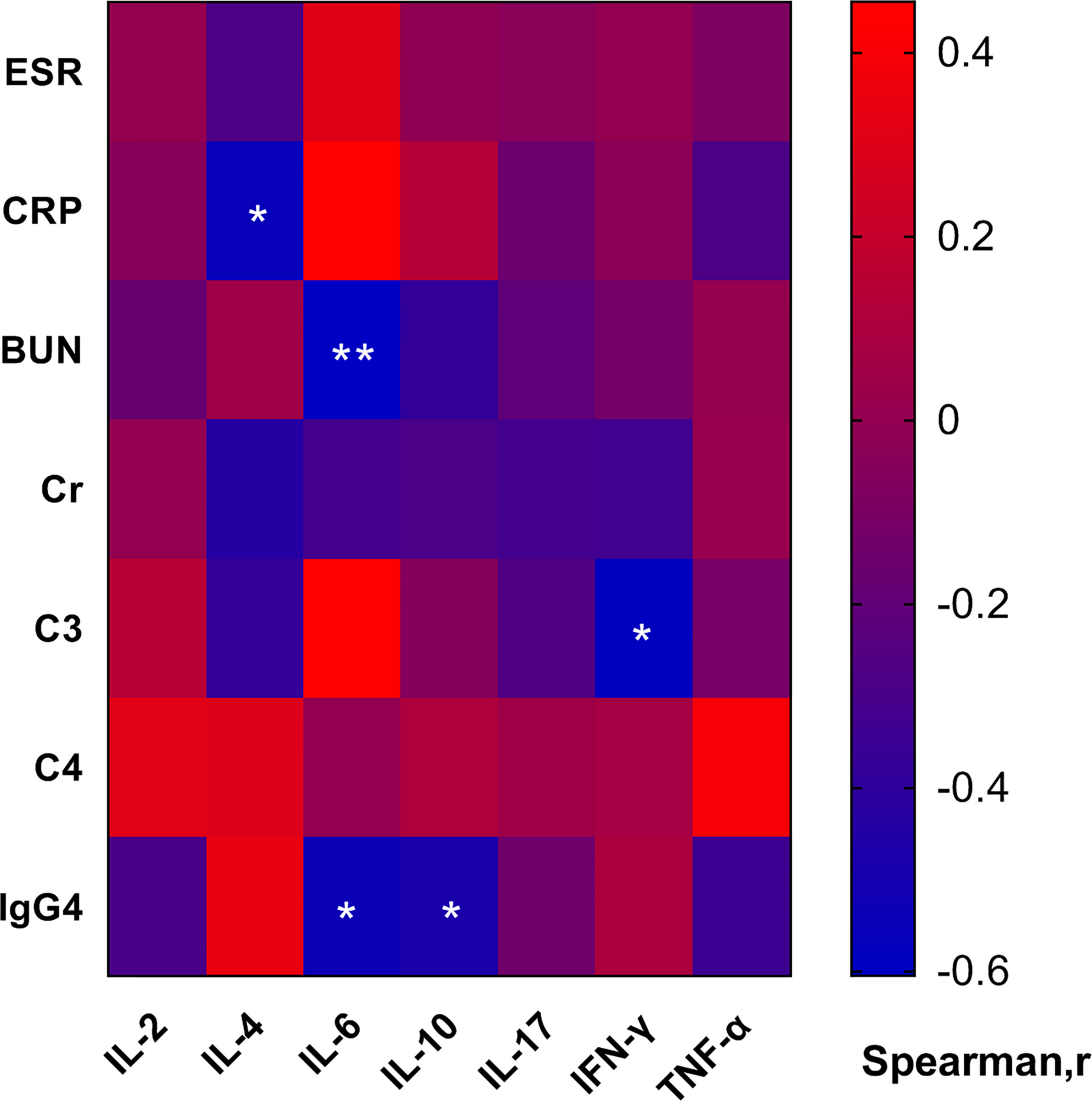
Heatmap of correlation of the serum cytokine levels with clinical and laboratory characteristics. In IRPF patients, IL-4 was negatively correlated with CRP, IL-6 was negatively correlated with BUN and IgG4, IFN-γ was negatively correlated with C3. (*p < 0.05, **p < 0.01).

IL-4 was negatively correlated with CRP (r=-0.560, p=0.016), but not with ESR. IL-6 was negatively correlated with blood urea nitrogen (BUN) (r=-0.605, p=0.008) and IgG4 (r= -0.536, p=0.022). IFN-γ was negatively correlated with C3 (r= -0.585, p=0.046) (**Figure 3**).

### 3.4 The absolute number of Treg cells was positively correlated with EOS

Absolute number of Treg cells were positively correlated with absolute values of EOS (r= 0.747, p<0.001) and negatively correlated with Tg-Ab (r= -0.546, p=0.023) in IRPF patients. Th17 cells were positively correlated with C3 (r= 0.560, p=0.030). Th2 cells were positively correlated with EOS (r=0.513, p=0.015) (**Table 4**). Th17 cells, Treg cells and Th17/Treg ratio were negatively correlated with IL-4, IL-6, IL-10, IL-17 IFN-γ, and TNF-α levels were not significantly correlated (p>0.05). (**Supplementary Table 3**)

Table 4Correlation analysis.

**Table:** 

	Th17 (cells/ml)		Th17%		Treg (cells/ml)		Treg%		Th17/Treg	
	r	p	r	p	r	p	r	p	r	p
ESR (mm/h)	-0.140	0.534	-0.225	0.314	0.002	0.994	0.133	0.555	0.012	0.959
CRP(mg/ml)	0.175	0.436	0.141	0.531	0.043	0.848	0.129	0.566	0.269	0.226
N(*10^9^/L)	0.155	0.490	0.183	0.416	-0.137	0.543	-0.255	0.253	0.278	0.211
EOS(*10^9^/L)	0.049	0.830	-0.232	0.299	0.747	<0.001***	0.309	0.162	-0.296	0.181
BUN(nmol/L)	-0.120	0.595	-0.028	0.903	-0.374	0.086	-0.277	0.211	-0.014	0.952
Cr(μmol/L)	-0.269	0.225	-0.337	0.125	0.150	0.506	0.043	0.848	-0.510	0.015*
C3(g/L)	0.560	0.030*	0.315	0.254	0.488	0.065	0.284	0.305	0.365	0.181
C4(g/L)	-0.178	0.525	-0.294	0.287	0.014	0.959	0.106	0.706	-0.130	0.645
Tg-Ab(IU/ml)	0.094	0.720	0.421	0.092	-0.546	0.023*	0.151	0.562	0.416	0.097

**Table:** 

			Th1(cells/ml)		Th1%		Th2 (cells/ml)		Th2%	
			r	p	r	p	r	p	r	p
ESR (mm/h)			-0.044	0.845	-0.185	0.410	0.009	0.970	-0.270	0.225
CRP(mg/ml)			-0.027	0.907	-0.194	0.388	0.006	0.978	0.003	0.990
N(*10^9^/L)			0.275	0.216	0.223	0.318	-0.016	0.942	0.176	0.434
EOS(*10^9^/L)			0.229	0.305	-0.454	0.034*	0.513	0.015*	-0.009	0.968
BUN(nmol/L)			-0.155	0.490	0.227	0.310	-0.219	0.328	0.141	0.531
Cr(μmol/L)			-0.165	0.462	-0.170	0.449	0.168	0.456	0.044	0.845
C3(g/L)			0.216	0.439	-0.250	0.368	0.474	0.075	0.344	0.210
C4(g/L)			0.061	0.828	0.153	0.586	-0.085	0.764	-0.432	0.108
Tg-Ab(IU/ml)			-0.132	0.613	0.479	0.052	-0.454	0.067	0.040	0.878

Statistics, Spearman correlation test.

Th1, T helper 1 cells; Th2, T helper 2 cells; Th17, T helper 17 cells; Treg, regulatory T cells; Th17/Treg, T helper 17 cell/regulatory T cell ratio; ESR, erythrocyte sedimentation rate; CRP, C-reactive protein; N, neutrophil; EOS, eosinophil; BUN, blood urea nitrogen; Cr, creatinine; C3, Complement 3; C4, Complement 4; Tg-Ab, thyroglobulin antibody.*P < 0.05, ***P < 0.001.

### 3.5 ROC curve analysis for predicting ureteral involvement in IRPF

**Figure 4:**
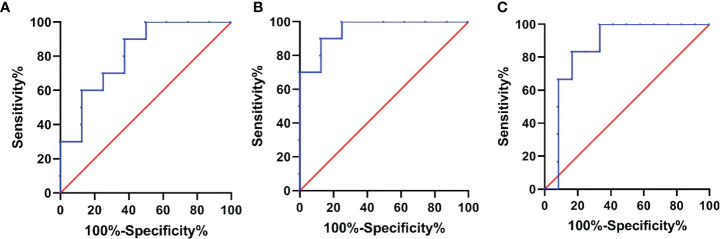
Receiver operating characteristic (ROC) curve of cytokines for predicting reteral involvement in IRPF. **(A)** Based on bilateral ureteral dilatation, the area under the ROC curve (AUC) of IL-10 was 0.813, sensitivity 0.900 and specificity 0.625. **(B)** Based on bilateral ureteral dilatation, the AUC of TNF-α was 0.950, sensitivity 0.900 and specificity 0.875. **(C)** Based on bilateral ureteral obstruction, the AUC of IL-6 was 0.861, sensitivity 0.833 and specificity 0.833.

According to the presence or absence of bilateral ureteral dilatation, the AUCs for IL-10 and TNF-α were 0.813 (sensitivity 0.900 and specificity 0.625, p=0.026) and 0.950 (sensitivity 0.900 and specificity 0.875, p=0.001), respectively. According to the presence or absence of bilateral ureteral obstruction, the AUC for IL-6 was 0.861 (sensitivity 0.833 and specificity 0.833, p=0.015).(**Figure 4**).

## 4 Discussion

This retrospective study analyzed the characteristics of peripheral blood lymphocytes, CD4+T cell subsets and cytokines in patients with IRPF and compared them with healthy people. Analysis showed that B cells in IRPF patients were significantly higher than HC. For CD4+T cell subsets, Treg cells were significantly lower in IRPF patients than HC, which may explain the significant increase in Th17/Treg. In addition, compared with HC, other cytokines except IL-2 were significantly increased in IRPF patients, and IL-6, IL-10, and TNF-α play some predictive value in whether IRPF involves the ureter. Our findings help to elucidate the pathogenesis of IRPF and provide new ideas for its assessment and treatment.

Our study found that compared with IgG4-RD-non-RPF, the levels of eosinophils, IgG4 and IgG4/IgG were significantly decreased, and the levels of neutrophils, C3 and C4 were increased in IRPF patients, which indicated that the pathogenesis of the two diseases was different. Wang et al. (14) conducted a similar study, which showed that the levels of eosinophils and IgG4 in IgG4-RD-RPF patients were higher than those in IRPF, suggesting that the pathogenesis of IgG4-RD-RPF and IRPF may be different. The study of Zen et al. (15) also confirmed this speculation again. In our cohort, the majority of patients were found to have elevated ESR and CRP (86.4% and 59.1%, respectively), which were significantly higher than those in the IgG4-RD-non-RPF group. ESR and CRP are usually used to monitor the clinical course of diseases, but they do not always reliably reflect disease activity (2).

Naïve CD4+T cells can differentiate into a variety of Th cell subsets and play various roles in autoimmune diseases. Th17 cells, a subset of CD4+T lymphocytes, produce inflammatory cytokines such as IL-6 and IL-17, which promote the body’s inflammatory response and are the main participants in autoimmune diseases. Treg cells, another important subpopulation of cells differentiated from naive CD4+T cells, secrete transforming growth factor–β (TGF-β) and IL-10, inhibit the production of inflammatory factors by macrophages and effector T cells, maintain tolerance and inhibit the production of autoimmune diseases. In recent years, the study of Th17/Treg immune balance has become more and more extensive. Studies have confirmed that Th17/Treg immune imbalance is the pathogenesis of a variety of autoimmune diseases, such as RA (Rheumatoid Arthritis) (16), SLE (Systemic Lupus Erythematosus) (17) and IBD (Inflammatory Bowel Disease) (18). However, there is no study on IRPF and Th cell subsets at present. Our study showed that there was no significant difference in Th17 cells in IRPF patients, while CD4+CD25+Foxp3+Treg cells were significantly reduced, leading to Th17/Treg immune imbalance and the pathogenesis of IRPF.

Th17 cells expressing the pro-inflammatory cytokine IL-17A have also been confirmed to promote fibrotic changes (19). Studies have shown that IL-17A is associated with the pathogenesis of various of fibrosis diseases, which may be related to neutrophil recruitment (20–23). IL-17A-driven fibrosis is inhibited by IL-10 and promoted by IFN-γ, and requires TGF-β, suggesting a synergistic role of IL-17A and TGF-β in the development of fibrosis. Moreover, some scholars have found that IL-17A can promote fibrosis by intensifying upstream inflammatory response and regulating downstream fibroblast activation, confirming that IL-1β – IL-17A – TGF-β1- cytokine axis is an important pathway of inflammation-driven fibrosis (24). CD4+CD25+Foxp3+Treg cells can secrete immunosuppressive cytokines IL-10 and TGF-β1. In the study of Kitani et al. (25), Treg cells can protect mice from TGF-β 1-mediated fibrosis by secreting IL-10. It is well known that TGF-β is one of the key drivers of fibrosis. Macrophage-derived TGF-β1 usually shows wound healing and pro-fibrotic activities, while TGF-β1 secreted by Treg cells can play anti-inflammatory and anti-fibrotic effects (25). However, some studies have proved that Treg cells secreting TGF-β can induce fibrosis (26). It is still not clear why Treg cells have anti-fibrosis activity in some cases and pro-fibrosis activity in other cases, which is a question worthy of further investigation. In addition, we also conducted multivariate logistic regression analysis, and the results showed that Th17/Treg ratio was independently correlated with IRPF, further confirming our speculation that Th17/Treg was involved in the pathogenesis of IRPF.

In addition, our study also observed that IL-4, IL-6, IL-10, IL-17, IFN-γ and TNF-α in IRPF patients were higher than those in HC, and IL-6, IL-10, and TNF-α were predictors of ureteral involvement by IRPF. IL-6 and IL-17 are proinflammatory cytokines secreted by Th17 cells. IL-6 promotes the activation of CD4+T cells and exacerbates inflammatory responses, and is an important promoter of Th17 differentiation. IL-6 antagonists can be used to treat chronic periaortitis (27). IL-17 is a very powerful inflammatory factor that can activate T cells and stimulate endothelial cells, epithelial cells and fibroblasts to induce inflammation. IL-17 also has a synergistic effect with TNF-α and up-regulates IL-6 expression to co-regulate inflammation. Studies have shown that TNF-α plays an important role in pulmonary interstitial fibrosis (28, 29). IL-10 is a representative anti-inflammatory cytokine, mainly secreted by Treg cells, which has a variety of immunomodulatory and inflammatory effects and plays an important role in maintaining immune homeostasis. IL-10 negatively regulates Th17 cells to increase the number of Treg cells (13). Studies have shown that in addition to anti-inflammatory effects, IL-10 can also promote the killing ability and memory formation of CD8+T cells, and promote the survival, proliferation and antibody production of B cells to play a pro-inflammatory role (30). The increase in IL-10 level compared with HC in this study may be caused by the pleiotropism of IL-10. In our cohort, absolute numbers of Th1, Th2, and Treg cells were reduced, but cytokine levels were all elevated, suggesting that other cells besides the CD4+ T cell subpopulation may be involved in the pathogenesis of IRPF. Future studies need to further evaluate the mechanisms of abnormal cytokine production in IRPF.

Recently, more and more studies have shown that fibroblasts play an important role in autoimmune diseases. IL-6 in retroperitoneal fibrous tissue stimulates the activation of B cells and fibroblasts, and B cells can also differentiate into plasma cells under the stimulation of IL-6, thus participating in the pathogenesis of IRPF (27). In addition, Th2 response-related chemokines such as CCL11/eosinophil activated chemokines are highly expressed in IRPF patients and contribute to eosinophil and mast cell recruitment. Eosinophils and mast cells express C-C-chemokine receptor 3(CCR3)(CCL11/eosinophils activated chemokine receptor) and stimulate fibroblast activation, proliferation, and collagen production (31). However, in the present study, Th2 cells and Treg cells were reduced and both were positively correlated with eosinophils, which may explain the phenomenon that eosinophils were not increased, but further studies with increased sample size are still needed. In addition, activated M2 macrophages can inhibit the production of extracellular matrix by fibroblasts, thereby inhibiting the fibrotic process (19).

IRPF usually exists in the retroperitoneal tissue and may involve multiple retroperitoneal organs, often causing ureteral obstruction and even renal failure. So far, the relationship between some organ involvement and CD4+T cell subsets has not been studied. Notably, our study found that Cr levels decreased with the increase of Th17/Treg in patients with IRPF, suggesting that Th17 and Treg may play a role in renal involvement. This is contrary to the study of Wang et al. (32), who found that higher Th17/Treg ratio and higher levels of Cr and BUN in ANCA-associated vasculitis may be associated with different pathogenesis among different diseases, and the specific mechanism of action remains to be further studied.

Although we have innovatively studied the relationship between IRPF and CD4+T cell subsets and cytokines, there are still limitations. First of all, IRPF is a rare disease, the number of medical records that can be collected is small, and some cases have some data missing. Secondly, this study was retrospective, so peripheral blood sample did not suffice for *in vitro* experiments. Third, there is no clear diagnostic standard for IRPF disease, which mainly relies on imaging diagnosis and pathological diagnosis. Although imaging diagnosis was performed on all patients in this study, only a few pathological data were collected, which was performed outside the hospital, and no report was found. Finally, direct evidence of the role of Th17 and Treg imbalance in the pathogenesis of IRPF needs to be confirmed by appropriate animal models.

## 5 Conclusion

Our study showed that the increased Th17/Treg ratio caused by the decrease of Treg cells in IRPF patients was related to the pathogenesis, but the precise mechanism of Th17 and Treg cells leading to IRPF was still not established. High levels of IL-4, IL-6, IL-10, IL-17, IFN-γ, and TNF-α may also contribute to the development of disease. It is necessary to further study on the cause of Treg reduction, which may be a potential target for treatment of IRPF and provide a new strategy for immunomodulatory therapy.

## Data availability statement

The raw data supporting the conclusions of this article will be made available by the authors, without undue reservation.

## Ethics statement

The studies involving human participants were reviewed and approved by Ethics Committee of the Second Hospital of Shanxi Medical University. The patients/participants provided their written informed consent to participate in this study.

## Author contributions

LL performed the data analyses and wrote the manuscript. HY, YW, YX participated in the collection of samples and clinical data. LJ, JZ participated in the performance of the research and statistical analysis. CG and XL participated in the study design and revising of the manuscript. CW provided intellectual input and supervision throughout the study and made a substantial contribution to manuscript drafting. All authors contributed to the article and approved the submitted version.

## Funding

This work was supported by the National Natural Science Foundation of China (No.81971543); Key Research and Development (R&D) Projects of Shanxi Province (201803D31119) and Four “Batches” Innovation Project of Invigorating Medical through Science and Technology of Shanxi Province(NO.2022XM05).

## Conflict of interest

The authors declare that the research was conducted in the absence of any commercial or financial relationships that could be construed as a potential conflict of interest.

## Publisher’s note

All claims expressed in this article are solely those of the authors and do not necessarily represent those of their affiliated organizations, or those of the publisher, the editors and the reviewers. Any product that may be evaluated in this article, or claim that may be made by its manufacturer, is not guaranteed or endorsed by the publisher.
