# Supplementary material for: Decreased absolute number of peripheral regulatory T cells in patients with idiopathic retroperitoneal fibrosis
Source: Front Immunol. 2022 Nov 29;13:1012513. doi: 10.3389/fimmu.2022.1012513 (PMC9744965; doi:10.3389/fimmu.2022.1012513)
Supplement: Supplementary file 1 [file Table_1.docx]

**Supplementary Material**

1. **Supplementary Tables**

**Table 1.** Absolute counts and proportions of peripheral blood lymphocytes in IRPF group(A), IgG4-RD-non-RPF(B) and Healthy control group(C).

| Cell count  (cells/μL) | IRPF(A)  (n=22) | IgG4-RD-non-RPF(B)  (n=36) | HC(C)  (n=28) | *P-*Value | A *vs.* B | *A vs. C* | *B vs. C* |
| --- | --- | --- | --- | --- | --- | --- | --- |
| Total T | 999.76  (678.68-1352.28) | 1251.92  (996.62-1775.63) | 1272.53  (1020.31-1619.83) | 0.026* | 0.036* | 0.071 | 1.000 |
| T% | 71.06  (65.46-74.05) | 72.81  (68.83-78.02) | 71.22  (64.92-75.00) | 0.203 | - | - | - |
| Total B | 124.08  (86.59-193.76) | 161.22  (122.61-278.95) | 212.50  (148.42-262.27) | 0.023* | 0.310 | 0.018* | 0.533 |
| B% | 10.00  (6.99-15.13) | 8.78  (6.64-14.14) | 10.27  (8.21-13.96) | 0.426 | - | - | - |
| NK | 215.16  (136.08-311.68) | 251.80  (177.12-303.19) | 290.83  (179.78-457.17) | 0.190 | - | - | - |
| NK% | 17.28  (11.08-20.44) | 12.97  (9.94-18.01) | 17.00  (11.15-21.00) | 0.169 | - | - | - |
| CD4+ | 562.77  (352.65-789.39) | 765.12  (564.34-1051.14) | 661.81  (578.51-759.50) | 0.015* | 0.011* | 0.246 | 0.754 |
| CD4+% | 40.31  (30.57-46.37) | 47.20  (38.46-51.38) | 39.36  (33.33-43.22) | 0.010* | 0.059 | 1.000 | 0.020* |
| CD8+ | 409.01  (249.52-572.14) | 435.39  (354.41-586.65) | 513.50  (357.26-645.59) | 0.441 | - | - | - |
| CD8+% | 26.54  (18.12-36.52) | 23.77  (19.16-28.91) | 26.50  (20.47-33.49) | 0.526 | - | - | - |

Results are expressed as the median and 25th and 75th percentiles.

Statistics: Kruskal-Wallis H test.

IRPF, Idiopathic retroperitoneal fibrosis; IgG4-RD-non-RPF, IgG4-related diseases without RPF; HC, Healthy control; Total T: total T cells; Total B: total B cells; CD4+: CD4+ T cells; CD8+: CD8+ T cell; NK: natural killer T cells. *P<0.05, **P<0.01, ***P<0.001

**Table 2**. Absolute counts and proportion of CD4+ T cells in IRPF group(A), IgG4-RD-non-RPF(B) and Healthy control group(C).

| Cell count  (cells/μL) | IRPF(A)  (n=22) | IgG4-RD-non-RPF(B)  (n=36) | HC(C)  (n=28) | *P*-Value | *A vs. B* | *A vs. C* | *B vs. C* |
| --- | --- | --- | --- | --- | --- | --- | --- |
| Th1 | 85.78  (55.58-135.96) | 158.04  (107.16-216.11) | 141.75  (82.73-159.18) | 0.001** | 0.001** | 0.118 | 0.348 |
| Th1% | 17.18  (9.98-24.12) | 19.50  (14.14-28.36) | 18.46  (14.50-23.99) | 0.533 | - | - | - |
| Th2 | 4.88  (2.87-7.68) | 8.33  (5.54-12.37) | 8.27  (4.93-10.66) | 0.005** | 0.004** | 0.043* | 1.000 |
| Th2% | 0.85  (0.70-1.23) | 1.02  (0.79-1.47) | 1.23  (0.87-1.67) | 0.123 | - | - | - |
| Th17 | 6.52  (4.79-9.18) | 7.97  (5.55-11.95) | 5.60  (3.16-8.78) | 0.032* | 0.731 | 0.682 | 0.027* |
| Th17% | 1.16  (0.82-2.03) | 1.16  (0.70-1.56) | 0.77  (0.45-1.07) | 0.011* | 0.957 | 0.012* | 0.090 |
| Treg | 19.55  (12.18-30.16) | 25.49  (19.80-43.60) | 34.55  (28.20-46.38) | 0.001** | 0.051 | <0.001*** | 0.225 |
| Treg% | 3.70  (2.85-4.50) | 3.52  (3.01-5.25) | 4.96  (4.21-6.30) | 0.001** | 1.000 | 0.003** | 0.005** |
| Th17/Treg% | 31.21  (24.00-46.82) | 31.68  (16.03-45.91) | 15.99  (8.93-22.56) | <0.001*** | 1.000 | <0.001*** | 0.001** |

Results are expressed as the median and 25th and 75th percentiles.

Statistics: Kruskal-Wallis H test.

IRPF, Idiopathic retroperitoneal fibrosis; IgG4-RD-non-RPF, IgG4-related diseases without RPF; HC, Healthy control; Th1: T helper 1 cells; Th2: T helper 2 cells; Th17: T helper 17 cells; Treg: regulatory T cells; Th17/Treg: T helper 17 cell/regulatory T cell ratio. *P<0.05, **P<0.01, ***P<0.001

**Table 3**. Correlation analysis of CD4+ T cells and cytokines.

|  | Th17 (cells/ml) | | | | Th17% | | | Treg (cells/ml) | | Treg% | | | Th17/Treg | | | |
| --- | --- | --- | --- | --- | --- | --- | --- | --- | --- | --- | --- | --- | --- | --- | --- | --- |
|  | r | | p | | r | | p | r | p | r | p | | r | | p | |
| IL-4 (pg/ml) | 0.013 | | 0.958 | | 0.204 | | 0.416 | -0.315 | 0.203 | 0.011 | 0.964 | | 0.119 | | 0.639 | |
| IL-6 (pg/ml) | 0.294 | | 0.236 | | -0.012 | | 0.961 | 0.191 | 0.428 | 0.086 | 0.735 | | 0.115 | | 0.651 | |
| IL-10 (pg/ml) | 0.084 | | 0.742 | | 0.160 | | 0.525 | -0.199 | 0.428 | 0.015 | 0.951 | | 0.131 | | 0.604 | |
| IL-17 (pg/ml) | 0.032 | | 0.900 | | 0.206 | | 0.413 | -0.179 | 0.478 | -0.082 | 0.748 | | 0.259 | | 0.299 | |
| IFN-γ(pg/ml) | -0.069 | | 0.785 | | 0.401 | | 0.099 | -0.327 | 0.185 | 0.042 | 0.868 | | 0.269 | | 0.280 | |
| TNF-α(pg/ml) | -0.002 | | 0.994 | | -0.206 | | 0.413 | -0.275 | 0.270 | -0.307 | 0.216 | | -0.049 | | 0.848 | |
|  | | Th1(cells/ml) | | | | Th1% | | | Th2 (cells/ml) | | | Th2% | | | |  |
|  |  | r | | p | | r | | p | r | p | | r | | p | |  |
| IL-4 (pg/ml) | | 0.011 | | 0.964 | | 0.286 | | 0.250 | -0.379 | 0.121 | | -0.192 | | 0.445 | |  |
| IL-6 (pg/ml) | | 0.408 | | 0.093 | | 0.162 | | 0.521 | 0.243 | 0.332 | | 0.109 | | 0.668 | |  |
| IL-10 (pg/ml) | | -0.018 | | 0.945 | | 0.238 | | 0.341 | -0.152 | 0.548 | | 0.030 | | 0.906 | |  |
| IL-17 (pg/ml) | | 0.086 | | 0.735 | | 0.212 | | 0.399 | -0.156 | 0.537 | | -0.150 | | 0.553 | |  |
| IFN-γ(pg/ml) | | -0.038 | | 0.880 | | 0.340 | | 0.168 | -0.645 | 0.004** | | -0.530 | | 0.024* | |  |
| TNF-α(pg/ml) | | 0.011 | | 0.964 | | 0.253 | | 0.311 | -0.031 | 0.903 | | 0.098 | | 0.700 | |  |

Statistics: Spearman correlation test.

Th1: T helper 1 cells; Th2: T helper 2 cells; Th17: T helper 17 cells; Treg: regulatory T cells; IL-4: interleukin-4; IL-6: interleukin-6; IL-10: interleukin-10; IL-17: interleukin-17; INF-γ: interferon-γ; TNF-α: tumor necrosis factor-α.*P<0.05, **P<0.01, ***P<0.001.
